# Temporal Trends of the Clinical, Resource Use and Outcome Attributes of ICU-Managed Candidemia Hospitalizations: A Population-Level Analysis

**DOI:** 10.14740/jocmr2484w

**Published:** 2016-02-27

**Authors:** Lavi Oud

**Affiliations:** Division of Pulmonary and Critical Care Medicine, Department of Internal Medicine, Texas Tech University Health Sciences Center at the Permian Basin, 701 W. 5th St., Odessa, TX 79763, USA. Email: lavi.oud@ttuhsc.edu

**Keywords:** Candidemia, Intensive care unit, Mortality, Organ failure, Outcomes

## Abstract

**Background:**

There are mixed findings on the longitudinal patterns of the incidence of intensive care unit (ICU)-managed candidemia, with scarcity of reports on the corresponding evolving patterns of patients’ clinical characteristics and outcomes. No population-level data were reported on the temporal trends of the attributes, care and outcomes of ICU-managed adults with candidemia.

**Methods:**

The Texas Inpatient Public Use Data File was used to identify hospitalizations aged 18 years or older with a diagnosis of candidemia and ICU admission (C-ICU hospitalizations) between 2001 and 2010. Temporal trends of the demographics, clinical features, use of healthcare resources, and short-term outcomes were examined. Average annual percent changes (AAPCs) were derived.

**Results:**

C-ICU hospitalizations (n = 7,552) became (AAPC) increasingly younger (age ≥ 65 years: -1.0%/year). The Charslon comorbidity index rose 4.2%/year, while the mean number of organ failures (OFs) increased by 8.2%/year, with a fast rise in the rate of those developing ≥ 3 OFs (+15.5%/year). Between 2001 and 2010, there was no significant change in utilization of mechanical ventilation and new hemodialysis among C-ICU hospitalizations with reported respiratory and renal failures (68.9% vs. 73.3%, P = 0.3653 and 15.5% vs. 21.8%, P = 0.8589, respectively). Hospital length of stay or total hospital charges remained unchanged during study period. Hospital mortality decreased between 2001 and 2010 from 39.3% to 23.8% (-5.2%/year). The majority of hospital survivors (61.6%) were discharged to another facility, and increasingly to long-term acute care hospitals, with routine home discharge decreasing to 11% by 2010.

**Conclusions:**

C-ICU hospitalizations demonstrated increasing comorbidity burden and rising development of OF, and matching rise in use of selected life-support interventions, though with unchanged in-hospital fiscal impact. There has been marked decrease in hospital mortality, but survivors had substantial residual morbidity with the majority discharged increasingly to another post-acute care facility.

## Introduction

Candidemia remains among the most common causes of bloodstream infections [1] and according to a recent multistate hospital survey in the United States (US), candida species have become the most common isolates in healthcare-associated bloodstream infections [2]. Predisposing risk factors for candidemia are commonly present in critically ill patients [3] and candidemic patients can require intensive care unit (ICU) care due to resultant critical illness [4], with candidemia remaining associated with high case fatality among the critically ill [5-8].

Only few studies have examined contemporary trends of the incidence of candidemia in ICU-managed patients, showing mixed findings, with unchanged [9] or a mix of rising with either preceding [8] or subsequently [10] plateauing incidence patterns. The sources of this variability are unclear. Data on the evolving patterns of the characteristics of critically ill candidemic patients can complement the observed epidemiological trends and may inform clinical practice and future studies of preventive and therapeutic interventions in this population.

However, although numerous studies have characterized critically ill candidemic patients, their care, and outcomes [4-6], there have been scarce data [7, 8] on the corresponding longitudinal patterns of these attributes.

We sought to examine the population-level temporal trends of the demographic, clinical, resource use, and short-term outcome characteristics of ICU-managed candidemic patients in Texas.

## Materials and Methods

### Setting and data sources

The Texas Inpatient Public Use Data File (TIPUDF) was used to perform a retrospective, population-based cohort study of ICU-managed adult state residents with a diagnosis of candidemia. The TIPUDF is an administrative dataset maintained by the Texas Department of State Health Services [11]. The use of the dataset has been previously described [12]. Briefly, TIPUDF includes detailed de-identified inpatient discharge data on the demographic, clinical, resource utilization, and outcome domains from state-licensed hospitals, and captures 93-97% of all hospital discharges in the state. The Institutional Review Board of Texas Tech Health Sciences Center has determined that the present study is exempt from formal review due to use of publicly available, de-identified data.

### Study population

We identified hospitalizations aged 18 years or older with a reported primary or secondary diagnosis of candidemia and with admission to ICU (termed C-ICU hospitalizations in the remainder of the manuscript) during the years 2001 - 2010. A diagnosis of candidemia was based on reported International Classification of Diseases, Ninth Revision, Clinical Modification (ICD-9-CM) code 112.5 [13, 14]. An admission to ICU was defined as presence of an ICU charge greater than $0.

### Data collection

We collected data on patients’ age, gender, race/ethnicity (categorized as non-Hispanic black (black), non-Hispanic white (white), Hispanic, and other), comorbid conditions (based on the Deyo modification of the Charlson comorbidity index [15]), medical or surgical hospitalization (based on diagnosis-related groups), type and number of failing organs [16, 17] (Supplementary Table 1, www.jocmr.org), life-support interventions (mechanical ventilation, central venous catheterization, and new hemodialysis; new hemodialysis was defined as a combination of a hemodialysis code and a code for acute renal failure) (Supplementary Table 2, www.jocmr.org), hospital length of stay, total hospital charges, and discharge disposition at the end of hospitalization. The categories of patients’ discharge disposition were grouped as death, hospice, home (with and without home health), another hospital, nursing facility, and other (leave against medical advice and unknown). Given the increasing use of hospice in severely ill hospitalized patients [17], those ending with death or discharge to hospice were further grouped as end-of-life (EOL) hospitalizations. Because patients with chronic critical illness are increasingly discharged to long-term acute care hospitals [18], we further examined use of these facilities among patients discharged to another hospital. Discharge destination (excluding EOL hospitalizations) was used as proxy of residual morbidity. Changes in the number of failing organs were used as a surrogate measure for severity of illness [17].

### Data analysis

Because TIPUDF provides discharge-level, rather than patient-level information, we reported identified ICU-managed events as number of hospitalizations.

The state of Texas masks gender data of hospitalizations with a diagnosis of infection with the human immunodeficiency virus (HIV), ethanol or drug abuse. Thus, analyses involving gender were restricted to hospitalizations without the aforementioned three diagnoses.

Because changes in the frequency of reported organ failures (OFs) over time may represent over-coding [16], we compared the rates of utilization of organ-specific life-support interventions among C-ICU hospitalizations with a specific OF (i.e., use of mechanical ventilation among hospitalizations with reported respiratory failure) at the start and end of study period.

Linear regression analyses of log-transformed data were used to examine the temporal trends of examined demographics, clinical attributes, use of healthcare resources, and outcomes, and to derive a corresponding relative average annual percent change (AAPC), using mean, median, and percentage data of the examined domains. Total hospital charges were adjusted to the consumer price index [19] (2010 dollars).

Group data are reported as numbers (percentages) for categorical variables and mean (standard deviation (SD)) or median (interquartile range (IQR)) for continuous variables, as appropriate. X^2^ tests were used to compare categorical data. Ninety-five percent confidence intervals (95% CIs) were calculated. We used SAS version 9.3 (SAS Institute, Cary, NC, USA) and MedCalc version 15.6 (MedCalc Software, Ostend, Belgium) software for data analyses. A two-sided P value < 0.05 was considered significant.

## Results

There were 7,552 C-ICU hospitalizations during study period. Details of the demographic characteristics and health insurance are outlined in Table 1. In addition, trends of use of various categories of health insurance are detailed in Table 2. Health insurance data were missing in one hospitalization. C-ICU hospitalizations became increasingly (AAPC (95% CI)) younger (age ≥ 65 years: -1%/year (-2.0% to -0.1%)), while their gender and race/ethnicity composition remained unchanged (data not shown). Medicare was the most common form of health insurance for the whole cohort and was used in 29.8% of C-ICU hospitalizations younger than 65 years. The health insurance used among C-ICU hospitalizations has changed between 2001 and 2010, with decreased use of Medicare (-12%) and marked rise in use of private insurance (+31%).

**Table 1:** The Demographics and Health Insurance Characteristics of C-ICU Hospitalizations, 2001 - 2010

Group	n = 7,552
Age (years), n (%)^a^	
18 - 44	1,177 (15.6)
45 - 64	2,774 (36.7)
≥ 65	3,601 (47.7)
Male, n (%)^a, b^	3,242 (46.5)
Race/ethnicity, n (%)^a^	
White	3,840 (50.8)
Hispanic	1,590 (21.1)
Black	1,512 (20.0)
Other	591 (7.8)
Missing	1 (0.3)
Health insurance, n (%)^a^	
Private	1,973 (26.1)
Medicare	4,212 (55.8)
Medicaid	770 (10.2)
Uninsured	478 (6.3)
Other	118 (1.6)
Missing	1 (0.3)

^a^Percent figures are rounded. ^b^Gender was masked in 575 hospitalizations. The denominator used to derive gender percentages for the cohort was based on hospitalizations with available gender designation (n = 6,977).

**Table 2:** Select Demographic Characteristics, Health Insurance, and Comorbidities of C-ICU Hospitalizations, 2001 - 2010

Group	2001 - 2002 (n = 905)	2003 - 2004 (n = 1,114)	2005 - 2006 (n = 1,752)	2007 - 2008 (n = 1,829)	2009 - 2010 (n = 1,952)	AAPC (95% CI)^a^	P-value
Male^b^ n, (%)	381 (45.7)	439 (42.3)	795 (49.8)	776 (46.1)	851 (47)	0.6 (-0.9 to 2.2)	0.3729
Age ≥ 65 years, n (%)	460 (50.8)	527 (47.3)	861 (49.1)	863 (47.2)	890 (45.6)	-1.0 (-2.0 to -0.1)	0.0382
Health insurance, n (%)							
Private	200 (22.1)	266 (23.9)	429 (24.5)	510 (27.9)	570 (29.2)	3.6 (2.3 to 5.0)	0.0002
Medicare	540 (59.7)	610 (54.8)	1,050 (59.9)	995 (54.4)	1017 (52.1)	-1.3 (-2.5 to -0.2)	0.0245
Medicaid	69 (7.6)	133 (11.9)	165 (9.4)	189 (10.3)	214 (11.0)	3.0 (-2.0 to 7.9)	0.2090
Uninsured	51 (5.6)	79 (7.1)	88 (5.0)	125 (6.8)	135 (6.9)	1.6 (-2.7 to 5.9)	0.4079
Other	45 (5.0)	25 (2.2)	22 (1.3)	10 (0.5)	16 (0.8)	-29.0 (-43.9 to -14.1)	0.0020
Charlson-Deyo comorbidity index^c^	2.3 (2.3)	2.1 (2.1)	3.0 (2.6)	3.2 (2.6)	2.9 (2.5)	4.2 (1.1 to 7.3)	0.0136
Comorbidities^d^, n (%)							
One or more comorbidity	699 (77.2)	858 (77.0)	1,494 (85.3)	1,577 (86.2)	1,653 (84.7)	1.4 (0.3 to 2.5)	0.0179
Myocardial infarction	55 (6.1)	62 (5.6)	141 (8.0)	171 (9.3)	190 (9.7)	7.1 (1.4 to 12.8)	0.0202
Congestive heart failure	222 (24.5)	289 (25.9)	643 (36.7)	584 (31.9)	519 (26.6)	1.6 (-2.7 to 5.90)	0.4177
Cerebrovascular disease	59 (6.5)	80 (7.2)	232 (13.2)	244 (13.3)	288 (14.8)	10.8 (5.8 to 15.8)	0.0011
Peripheral vascular disease	55 (6.1)	61 (5.5)	164 (9.4)	252 (13.8)	245 (12.6)	11.3 (4.8 to 17.8)	0.0038
Chronic lung disease	192 (21.2)	225 (20.2)	491 (28.0)	438 (23.9)	414 (21.2)	0.6 (-3.1 to 4.4)	0.7102
Connective tissue disease	23 (2.5)	32 (2.9)	51 (2.9)	64 (3.5)	67 (3.4)	3.3 (-1.1 to -7.7)	0.1198
Diabetes mellitus	121 (13.4)	124 (11.1)	429 (24.5)	545 (29.8)	520 (26.6)	11.3 (4.0 to 18.6)	0.0071
Renal disease	136 (15.0)	194 (17.4)	491 (28)	570 (31.2)	555 (28.4)	9.4 (5.2 to 13.6)	0.0009
Liver disease	73 (8.1)	90 (8.1)	217 (12.4)	256 (14.0)	311 (15.9)	9.1 (4.7 to 13.4)	0.0014
Malignancy	162 (17.9)	177 (15.9)	302 (17.2)	319 (17.4)	271 (13.9)	-1.6 (-5.0 to 1.7)	0.2971

^a^AAPC: average annual percent change (95% confidence intervals). ^b^The denominator used to derive male percentage for the cohort was based on hospitalizations with available gender designation. ^c^Mean (standard deviation). ^d^Based on the Charlson-Deyo comorbidity index.

Comorbid conditions were reported in the majority of C-ICU hospitalizations (Table 2), and were increasingly present, with the mean (SD) Charlson-Deyo comorbidity index rising 4.2%/year (1.1-7.3%). Congestive heart failure and renal disease were the most commonly reported conditions among examined comorbidities, with the fastest growth from 2001 to 2010 noted in renal failure (123%) and cerebrovascular disease (114%). HIV infection was rarely reported (132 (1.7%) hospitalizations). Surgical C-ICU hospitalizations (n = 4,042, 53.5%) progressively decreased (-1.4%/year (-2.1% to -0.7%)) over study period.

The patterns of OF and used healthcare resources are detailed in Table 3. The mean number of failing organs rose 8.1%/year (5.2-10.9%), with the growth rate of those with ≥ 3 organ failures increasing 15.5%/year (9.2-21.7%). The rates of all examined OFs increased over study period, with those affecting the respiratory, cardiovascular and renal systems remaining the most common. The fastest growth rate of individual OFs between 2001 and 2010 involved the neurological (469%) and cardiovascular (215%) systems.

**Table 3:** Organ Failure and Healthcare Resource Utilization Patterns of C-ICU Hospitalizations, 2001 - 2010

Group	2001 - 2002 (n = 905)	2003 - 2004 (n = 1,114)	2005 - 2006 (n = 1,752)	2007 - 2008 (n = 1,829)	2009 - 2010 (n = 1,952)	AAPC (95% CI)^a^	P-value
Organ failure^b^	1.4 (1.1)	1.3 (1.1)	2.0 (1.5)	2.2 (1.6)	2.4 (1.6)	8.1 (5.2 to 10.9)	0.0002
Any organ failure (%)	684 (75.6)	818 (73.4)	1,473 (84.1)	1,585 (86.7)	1,707 (87.4)	2.3 (1.4 to 3.3)	0.0005
Three or more organ failures (%)	132 (14.6)	155 (13.9)	603 (34.4)	673 (36.8)	844 (43.2)	15.5 (9.2 to 21.7)	0.0004
Respiratory (%)	516 (57.0)	602 (54.0)	1,101 (62.8)	1,168 (63.9)	1,273 (65.2)	2.1 (0.9 to 3.3)	0.0039
Cardiovascular (%)	156 (17.2)	200 (18.0)	590 (33.7)	692 (37.8)	779 (39.9)	12.4 (7.9 to 16.9)	0.0002
Renal (%)	270 (29.8)	306 (27.5)	696 (39.7)	811 (44.3)	994 (50.9)	7.5 (4.8 to 10.30	0.0002
Hepatic (%)	33 (3.6)	34 (3.1)	112 (6.4)	149 (8.1)	182 (9.3)	13.9 (6.7 to 21.0)	0.0021
Hematological (%)	117 (12.3)	155 (13.9)	416 (23.7)	446 (24.4)	454 (23.3)	8.7 (4.2 to 13.10	0.0020
Metabolic (%)	87 (9.6)	94 (8.4)	303 (17.3)	355 (19.4)	366 (18.8)	10.8 (4.9 to 16.6)	0.0028
Neurological (%)	47 (5.2)	40 (3.6)	272 (15.5)	368 (20.1)	543 (27.8)	24.9 (14.5 to 35.3)	0.0006
Mechanical ventilation (%)	368 (40.7)	427 (38.3)	839 (47.9)	885 (48.4)	905 (46.4)	2.5 (0.6 to 4.4)	0.0155
New hemodialysis (%)	40 (4.4)	48 (4.3 )	177 (10.1)	205 (11.2)	220 (11.3)	13.7 (6.6 to 20.7)	0.0021
Central venous catheterization (%)	422 (46.6)	534 (47.9)	1,082 (61.8)	1,136 (62.1)	1,237 (63.4)	4.3 (2.6 to 6.1)	0.0004
Hospital length of stay (days)^b^	33.2 (27.8)	31.2 (23.8)	33.0 (26.6)	33.4 (31.7)	32.2 (32.1)	-0.1 (-1.0 to 0.9)	0.9226
Total hospital charges (dollars)^c^	226,997 (108,200 - 470,594)	218,094 (115,397 - 401,271)	227,336 (126,166 - 393,787)	235,205 (123,707 - 400,408)	235,457 (120,512 - 433,173)	0.8 (-0.1 to 1.8)	0.0720

^a^AAPC: average annual percent change (95% confidence intervals). ^b^Mean (standard deviation). ^c^Median (interquartile range); hospital charges are adjusted for inflation (2010 dollars).

The use of examined life-support interventions increased during study period (Table 3). The use of mechanical ventilation and new hemodialysis among those with specific OFs tended to rise but was not statistically different between 2001 and 2010: 1) mechanical ventilation: 68.9% vs. 73.3% (P = 0.3653) among those with respiratory failure; 2) new hemodialysis: 15.5% vs. 21.8% (P = 0.8589) among those with acute renal failure. The mean (SD) hospital length of stay (32.7 (29.2) days) remained unchanged, while the median (IQR) total hospital charges ($231,141 (120,093 - 416,028)) tended to slowly rise (though not statistically significantly) during study period.

Discharge dispositions of C-ICU hospitalizations are detailed in Table 4. Hospital mortality was 29.8% for the whole cohort and decreased by 5.2%/year (-7.4% to -3.0%), while discharge to hospice rose 24%/year (10.6-37.5%). Among hospital survivors (excluding those discharged to hospice), 61.6% were discharged to another facility. Transfers to a long-term acute care hospital rose 11.9%/year (6.4-17.5%) since 2003, accounting for 20.9% of all discharges in 2010. Routine home discharge declined by 6.5%/year (-10.0% to -3.0%), reaching 11% of all discharges in 2010.

**Table 4:** Discharge Disposition of C-ICU Hospitalizations, 2001 - 2010

Group (%)^b^	2001 - 2002 (n = 905)	2003 - 2004 (n = 1,114)	2005 - 2006 (n = 1,752)	2007 - 2008 (n = 1,829)	2009 - 2010 (n = 1,952)	AAPC (95% CI)^a^	P-value
End-of-life hospitalizations^c^	367 (40.6)	381 (34.2)	681 (38.9)	641 (35.0)	617 (31.6)	-2.4 (-4.5 to -0.2)	0.0349
Death	355 (39.2)	342 (30.7)	569 (32.5)	500 (27.3)	487 (24.9)	-5.2 (-7.4 to -3.0)	0.0006
Hospice	12 (1.3)	39 (3.5)	112 (6.4)	141 (7.7)	130 (6.7)	24.0 (10.6 to 37.5)	0.0033
Home	241 (26.6)	338 (30.3)	390 (22.3)	443 (24.2)	453 (23.2)	-2.5 (-5.2 to 0.2)	0.0667
Routine home	178 (19.7)	187 (16.8)	201 (11.5)	230 (12.6)	230 (11.8)	-6.5 (-10.0 to -3.0)	0.0025
Home health care	63 (7.0)	151 (13.6)	189 (10.8)	213 (11.6)	223 (11.4)	3.9 (-1.9 to 9.7)	0.1566
Another hospital	111 (12.3)	250 (22.4)	440 (25.1)	490 (26.8)	578 (29.6)	9.5 (2.5 to 16.5)	0.0143
Long-term care hospital^d^	-	123 (11.0)	208 (11.9)	349 (19.1)	391 (20.0)	11.9 (6.4 to 17.5)	0.0019
Nursing facility	138 (15.2)	138 (12.4)	233 (13.3)	245 (13.4)	286 (14.7)	0.2 (-3.0 to 3.4)	0.9081
Other^e^	48 (5.3)	7 (0.6)	8 (0.5)	10 (0.5)	18 (0.9)	-11.3 (-34.8 to 12.1)	0.2972

^a^AAPC: average annual percent change (95% confidence interval). ^b^Percent figures are rounded. ^c^End-of-life hospitalizations were those with either hospital death or discharge to hospice. ^d^Examination of annual changes was limited to the years 2003 - 2010, as the first discharges to a long-term care facility were reported in 2003. ^e^Leave against medical advice and unknown discharge destination.

## Discussion

We found that over the last decade, there has been increasing burden of comorbidity, rising development of OF, and corresponding increased use of the examined life-support interventions among C-ICU hospitalizations. These trends were associated unexpectedly with substantial progressive decrease in hospital mortality. However, the majority of hospital survivors were discharged to another facility and there has been rapid increase in transfers to long-term acute care hospitals.

To our knowledge, the present study represents the first population-level examination of the temporal trends of the demographic, clinical, resource use, and outcome attributes of ICU-managed candidemic patients in the US, and the largest cohort to date in this population. Two recent studies of critically ill candidemic patients in Brazil [7] and France [8] have examined changes in patients attributes between two 4-year periods [7] and trends of annual 30-day mortality and antifungal therapy [8]. However, other multi-year ICU-focused studies of candidemia did not examine evolving patterns of patient attributes, likely in part due to challenges posed by relatively small cohort size [6, 20], thus affecting the interpretation of the present findings in the context of prior work.

Reported gender composition varied in prior studies [4-6, 14]. Our findings of female predominance among C-ICU hospitalizations are in agreement with a national population-based study in the US by Zaoutis and colleagues [14]. However, candidemic patients were mostly male in population-level studies in other countries [21, 22] and in reports on active surveillance registries in the US [23]. Although sepsis in general is considered to develop more commonly among males [24], the role of gender in development of candidemia requires further study.

C-ICU hospitalizations have been increasingly younger, in contrast to an unchanged age composition in other studies [7, 8]. The sources of the discordant trends are unclear, but may be related to differences in population mix due to geographical variation and that between tertiary care hospitals and broader array of acute care facilities in a population-based study.

Black and Hispanic patients were represented among C-ICU hospitalizations in substantially higher and lower rates, respectively, as compared to their share in the adult population in Texas [25]. Our findings are in agreement with prior reports showing markedly higher rates of candidemia in black patients [3], and with the findings by Zaoutis and colleagues, showing that adult Hispanic patients are underrepresented as compared to their national population share in the US [14]. However, the impact of Hispanic ethnicity on development of candidemia in the US population has not been systematically examined [3] and Hispanic patients were often not considered as a specific group in US studies [23, 26, 27]. Nevertheless, a report by Barnatto and colleagues showed an adjusted lower risk of severe sepsis among Hispanics in the US [28]. However, as noted by the investigators, administrative population-level studies cannot distinguish between differences in biological susceptibility and residual cofounding [28].

The health insurance used by candidemic patients has not been previously reported, though risk of sepsis-associated hospitalizations and related mortality [29], as well as processes of care among critically patients [30] has been reported to vary in association with specific health insurance categories. Our finding that nearly one-third of C-ICU hospitalizations younger than 65 years had Medicare insurance further underscores the frequent occurrence of substantial comorbidity burden in this cohort. The marked decrease in use of Medicare insurance over time was likely, for the most part, due to near-similar absolute decrease in elderly C-ICU hospitalizations over the past decade. However, the sources of the predominant increase of C-ICU hospitalizations with private insurance vs. other alternatives are unclear.

The findings of the most prevalent comorbidities affecting C-ICU hospitalizations contrast with common reports of malignancy being the most common comorbidity and were reported at higher rates than in the present study [5, 6, 8, 27]. The sources of the difference are unclear, but may be related to common studies involving referral centers [7, 8, 27] or academic hospitals [5]. In addition, previous studies provided at times limited data on categories of comorbid conditions [4, 6, 8], and occurrence of heart failure (the most common comorbidity in the present study) was rarely reported [27]. Similarly, the frequency of overall occurrence of comorbidities among candidemic critically ill patients was generally not described [5-8, 20, 27]. However, our finding that the majority of C-ICU hospitalizations had one or more of the examined comorbidities is in agreement with a recent study by Ylipalosaari and colleagues [4]. Finally, the increasing burden of comorbid conditions among C-ICU hospitalizations in the present cohort likely reflects overall increasing occurrence of chronic illness in the US population [31] and among the critically ill [17, 32].

Data on individual OFs were reported infrequently in studies of critically ill candidemic patients [27, 33, 34], with the most commonly affected systems being respiratory, cardiovascular, and renal, similar to the present study. The substantial rise in the reported number of failing organs and rates of individual OFs may represent increased clinician awareness, better documentation, or possible over-coding [16], especially given the progressive decrease in hospital mortality in the present cohort. However, the aforementioned changes were associated with corresponding unchanged rates of use of system-specific life-support interventions (i.e., mechanical ventilation among hospitalizations with respiratory failure), coupled with increased discharge to hospice, decreased rates of routine home discharge, and rapid rise of transfers to long-term acute care hospitals. These findings suggest that the rising occurrence of OF may reflect actual increase in severity of illness in the present cohort. The causes of an actual rise in the number of failing organs among D-ICU hospitalizations are unclear, but may be related in part to the increasing burden of comorbidities, which has been reported to be associated with development of OF [35]. Our findings contrast those of a recent study by Colombo and colleagues, reporting statistically insignificant downtrends of severity of illness scores in critically ill candidemic patients [7]. The source of difference is unclear, but may be related in part to different population mix in different geographical areas [36], the noted restriction to tertiary centers, and to the mostly unchanged comorbidity burden in the latter study [7].

The findings of lengthy hospital stay among candidemic patients are in agreement with prior reports [4, 5, 33]. There have not been, to our knowledge, studies on the fiscal impact associated with candidemia in critically ill patients, though studies not restricted to ICU populations showed, as expected, substantial excess costs [3, 14]. The total hospital charges in the present cohort make C-ICU hospitalizations the most expensive condition among hospitalizations in Texas [37].

The lack of significant change in hospital length of stay and total hospital charges among increasingly sick patients can imply marked improvement in care efficiencies. However, it is likely that there has been incremental shift of inpatient length of stay and fiscal burden in the present cohort to post-acute care facilities, as shown by the rapidly rising transfers to long-term acute care hospitals. These changes in discharge patterns reflect broader changes in the US, related to increasing fiscal pressures on short-term hospital [38].

Prior reports on short-term outcomes of ICU-managed candidemia focused on patients’ mortality [4-8]. The present study complements and extends available data, showing prevalent adverse impact of candidemia on hospital survivors. Our findings suggest substantial and incremental residual morbidity of C-ICU hospitalizations, with only one in 10 having routine home discharge by 2010, and with the majority discharged to another facility, with likely rising occurrence of chronic critical illness among hospital survivors, as evidenced by the noted incremental transfer to long-term acute care hospitals.

The present study describes the first substantial and progressive population-level decrease in hospital mortality and EOL hospitalizations of candidemic critically ill patients. These changes occurred despite an increasingly sick cohort and may reflect in part the impact of improving patient care [39]. Similar findings of decreasing hospital mortality among patients with rising burden of chronic illness and that of OF were described in with severe sepsis [17]. In addition, decreasing mortality in the present cohort was also likely related to both the increasingly younger C-ICU hospitalizations and rising use of private health insurance, as the latter was shown to be associated with the lowest risk of death, as compared to other sources of health insurance (or lack thereof) [29, 30]. Hospital mortality in the present cohort was overall lower than commonly reported mortality data in critically ill candidemic patients [4-8]. The difference may reflect in part reporting of mortality at a pre-specified day number (i.e., 30-day mortality) [7, 8], as well as international outcome variability among critically ill populations, related to differences in population mix and practice patterns [5, 36]. However, part of the difference may be related to possibly sicker populations managed in tertiary [7, 8] and predominantly academic centers [5].

Nevertheless, it is likely that the favorable changes of in-hospital death underestimate short-term mortality of C-ICU hospitalizations, with the former possibly biased by the rapid rise in transfers to long-term acute care hospitals, as a high rate of early death has been reported among the latter [18]. Similar concerns were raised about possible bias in findings of decreasing hospital morality among critically ill patients in the general population, considered by other investigators to be related in part to increasing shift of hospital deaths to post-acute care facilities [32], in association with the noted fiscal pressures on US hospitals [38] and use of hospital mortality as a performance measure [40, 41].

Previous studies of changes in mortality of critically ill candidemic patients described mixed findings, with reported decreasing [7] and rising [8] 30-day mortality. The conflicting results are difficult to reconcile as, in contrast to the present study, the investigators reported no significant change in age [7, 8], most reported comorbidities, severity of illness scores [7], or “major characteristics” [8], and with both studies reporting increased use of echinocandins [7, 8]. Our findings are in agreement with studies in the US that did not focus on ICU patients, showing large decreases in 30-day mortality in two metropolitan areas [23] and at a single-center setting [42]. However, in contrast to our study, there was decrease in the burden of comorbidities in one study [42] and drop in occurrence of malignancy in the other [23]. None of the aforementioned four studies provided data on non-mortality outcomes.

The study has several limitations. We used a retrospective design with its attendant limitations. In addition, administrative data provide limited clinical detail and we could not distinguish ICU-acquired vs. non-ICU acquired candidemia, though both patient categories were often described as a single group [5, 6, 27, 33]. However, population-level data can complement and transcend the well-described regional and local variability in available critical care resources [43], rates of ICU utilization [44, 45], processes of care [45, 46], organization [46], and discharge practices [40, 41], and thus can provide broader perspective on examined conditions.

Although we used similar ICD-9 code-based approach to that of other investigators [13, 14], it is possible that some hospitalizations have been misclassified. The sensitivity and specificity of the code for disseminated candidiasis in identifying candidemia in administrative datasets is unknown and it is possible that we underestimated the burden of candidemia [14]. However, it is unlikely that the classification limitations biased the observed temporal trends.

In addition, established severity of illness scores cannot be derived from administrative data and we used the number of failing organs as a surrogate measure of changes in severity of illness over time. However, similar approach was employed by other investigators [17], as the number of failing organs remains associated with incremental risk of death among critically ill patients [17, 47].

Because the state of Texas does not provide tools to convert hospital charges to costs, we reported hospital charges rather than costs of care, limiting comparisons with other cost data.

Finally, although we examined a cohort of C-ICU hospitalizations in a large state with a diverse population, the characteristics of patients admitted to ICU, the used resources, and short-term outcomes may vary across states and nationally.

In conclusion, over the past decade ICU-managed candidemic hospitalizations in Texas became increasingly sick, with rising burden of comorbidities and increasing severity of illness, and required substantial, though unchanged, use of health care resources. ICU-managed candidemia became progressively less fatal, though changes in short-term mortality may have been underestimated. Hospital survivors incurred increasing residual morbidity, with the majority transferred to other facilities, and only one in 10 had routine home discharge by 2010. Additional studies in other populations and healthcare environments are warranted to examine the contemporary changes among critically ill candidemic patients.
